# Omega-3 polyunsaturated fatty acids and cardiac rhythm: an introduction

**DOI:** 10.3389/fphys.2012.00457

**Published:** 2012-12-06

**Authors:** George E. Billman

**Affiliations:** Department of Physiology and Cell Biology, The Ohio State UniversityColumbus, OH, USA

“Sine doctrina, vita est quasi mortis imago” [Without, education, life is but the image of death] Dionysius Cato (Roman author, Fl. 4th c. AD).

“If I can stop one heart from breaking, I shall not live in vain” Emily Dickinson (American poet, 1830–1886).

The effective management of cardiac arrhythmias, either of atrial or of ventricular origin, remains a major challenge for the cardiologist. Sudden cardiac death most frequently due to ventricular tachyarrhythmias (Hinkle and Thaler, 1982; Bayes de Luna et al., 1989; Greene, 1990) remains the leading cause of death in industrially developed countries, accounting for between 300,000 and 500,000 deaths each year in the United States (Abildstrom et al., 1999; Zheng et al., 2001). In a similar manner, atrial fibrillation is the most common rhythm disorder (Kannel et al., 1998; Lakshminarayan et al., 2006), accounting for about 2.3 million cases in the United States and has been projected to increase by 2.5-fold over the next half century (Anonymous, 1998). Indeed, the prevalence of this arrhythmia increases with each decade of life (0.5% patient population between the ages of 50 and 59 years climbing to almost 9% at age 80–89 years) and contributes to approximately one-quarter of ischemic strokes in the elderly population (Kannel et al., 1998; Lakshminarayan et al., 2006). The economic impact associated with the morbidity and mortality resulting from cardiac arrhythmias is enormous [incremental cost per quality-adjusted life-year as much as US $558,000 (Byrant et al., 2005)].

Despite the enormity of this problem, the development of safe and effective anti-arrhythmic agents remains elusive. Several anti-arrhythmic drugs have actually been shown to increase, rather than to decrease, the risk for arrhythmic death in patients recovering from myocardial infarction (Echt et al., 1991; Waldo et al., 1996) while even “optimal” pharmacological therapy fails to suppress these arrhythmias completely (Buxton et al., 1999). For example, the one-year mortality is 10% or higher, with sudden death accounting for approximately one-third of the deaths, in post-myocardial infarction patients treated with β-adrenergic receptor antagonists (Buxton et al., 1999). Implantable cardioverter defibrillators (ICDs) have been shown to reduce cardiac mortality, providing a better protection from sudden death than current pharmacological therapy in certain high-risk patient populations (Buxton et al., 1999; Connelly et al., 2000). However, these devices are expensive to use and maintain (Groeneveld et al., 2006), negatively affect the patient's quality of life (Groeneveld et al., 2006), have a significant risk for inappropriate shock delivery (Poole et al., 2008), are ineffective in females patients (Henyan et al., 2006), and, perhaps most importantly, only extend life by a mean of 4.4 months (Connelly et al., 2000). Given the adverse outcomes associated with ICDs and many anti-arrhythmic medications, as well as the partial protection afforded by even the best agents (e.g., β-adrenergic receptor antagonists and ICDs), it is obvious that more effective anti-arrrhythmic therapies must be developed.

The cardiovascular benefits of dietary omega-3 polyunsaturated fatty acids (n-3 PUFA) have been actively investigated for nearly 40 years. Beginning with the pioneering studies of Bang and Dyerberg (Dyerberg et al., 1978; Bang et al., 1980), epidemiological data provide strong evidence for an inverse relationship between fatty fish consumption and cardiac mortality (Kromhout et al., 1985; Daviglus et al., 1997). In contrast to these observational studies, interventional studies using n-3 PUFAs for the secondary prevention of adverse cardiovascular events in patients with heart disease have yielded conflicting results. Some studies have reported reduced sudden cardiac death or mortality (Burr et al., 1989; Marchioli et al., 2002), while other more recent studies have reported that n-3 PUFAs either had no effect on cardiac arrhythmias [either ventricular arrhythmias/sudden death (Brouwer et al., 2006; Yokoyama et al., 2007; GISSI-HF Investigators, 2008; Kromhout et al., 2010; Rauch et al., 2010) or atrial fibrillation (Kowey et al., 2010; Mozaffarian et al., 2012; Sandesara et al., 2012)] or actually increased adverse cardiac events (Burr et al., 2003; Raitt et al., 2005). Not surprisingly, meta-analysis of these studies have yielded similar conflicting results (Hooper et al., 2004; Jenkins et al., 2008; Brouwer et al., 2009; Leon et al., 2009; Zhao et al., 2009; Filion et al., 2010) with the most recent study finding that omega-3 fatty acids were neutral, neither increasing nor decreasing the risk for arrhythmias (Rizos et al., 2012). Similar conflicting results have been obtained from animals models (McLennan et al., 1988; Billman et al., 1994; Coronel et al., 2007; Billman et al., 2012). Of particular note, dietary n-3 PUFAs increased rather than decreased susceptibility to arrhythmias induced by regional myocardial ischemia in isolated hearts (Coronel et al., 2007) and provoked ventricular fibrillation in conscious animals previously shown to be at a low risk for malignant arrhythmias (Billman et al., 2012). Despite these inconsistent findings, the American Heart Association and the American College of Cardiology continue to recommend fish oils for the secondary prevention of coronary artery disease (Kris-Etherton et al., 2003; Smith et al., 2006). Based in part upon these recommendations, consumer demand for n-3 PUFA products (both nutritional supplements and foods enriched with these lipids) has exploded. It has been estimated that 5–10% of the adult US population use fish oil supplements and sales are projected to exceed 7 billion dollars by the end of 2011 [www.marketresearch.com, product reports].

Despite the intensive marketing of fish oil products, a scientific consensus on the effects of n-3 PUFA on cardiac rhythm has yet to be reached. It is the purpose of this book to stimulate a discussion on the putative benefits of n-3 PUFAs on cardiac rhythm. The book contains both state-of-the art reviews of the literature and original research articles that address various aspects of the effects of n-3 PUFAs on cardiac rhythm. The book is divided into three sections. The first section addresses the effects of n-3 PUFAs on heart rate variability (chapters 2–4). The second section provides comprehensive reviews of the effects of n-3 PUFAs on ventricular arrhythmias/sudden death (chapters 5–8) and on atrial fibrillation (chapters 8–10). The third and final section (chapters 11–16) evaluates the cellular mechanisms by which n-3 PUFAs can influence arrhythmia formation. By understanding how n-3 PUFAs affect the cardiac rhythm, the author hopes that this brief monograph will provide an education sufficient to keep at least one heart from breaking.
